# A novel open synovectomy technique using an arthroscopy shaver blade in revision surgery to treat infected total knee arthroplasty: a technical note

**DOI:** 10.1186/s13018-022-03024-5

**Published:** 2022-03-03

**Authors:** Oog-Jin Shon, In Jun Lee, Gi Beom Kim

**Affiliations:** 1 Present Address: Department of Orthopedic Surgery, Yeungnam University College of Medicine, 170 Hyeonchung-ro Nam-gu, Daegu, 42415 Republic of Korea; 2 Department of Orthopedic Surgery, Yeungnam University Medical Center, 170 Hyeonchung-ro Nam-gu, Daegu, 42415 Republic of Korea

**Keywords:** Infected total knee arthroplasty, Second-stage revision surgery, Synovectomy, Arthroscopic shaver blade

## Abstract

**Background:**

This article introduces a novel open synovectomy technique using an arthroscopy shaver blade to effectively remove intra-articular synovitis during revision surgery for infected primary total knee arthroplasty.

**Methods (techniques):**

Open synovectomy is performed using a 4.2-mm arthroscopy shaver blade, and the handpiece is connected to suction drainage. Suction is supplied through the central cylinder of the shaver blade to bring the debrided fragments of soft tissue into the window. Grossly inflamed, reddened, diseased synovium is debrided to reveal yellowish, healthy synovium. The inflamed tissues of the knee joint (suprapatellar pouch, medial and lateral gutters, and peripatellar area) are debrided. Then, with maintaining full flexion of the knee joint, a shaver equipped with a longer bar can be used to easily access the medial and lateral posterior compartments, which are generally difficult to access.

**Results:**

During a mean of 13.5-month follow-up, there was no recurrent infection in either group; however, patients who underwent the novel technique improved significantly faster in terms of acute serological markers during the first period.

**Conclusions:**

This technique yielded favorable outcomes compared with the conventional technique. In particular, it may facilitate the approach to the posterior joint space, which is difficult to access.

**Supplementary Information:**

The online version contains supplementary material available at 10.1186/s13018-022-03024-5.

## Introduction

The number of total knee arthroplasties (TKAs) has increased with the growing elderly population, which has inevitably resulted in a corresponding increase in the prevalence of postoperative infection [1, 2]. Infection following TKA is a serious condition that can lead to many complications, and its management is often challenging [3–5]. Despite advances in diagnostic tools and treatment strategies, the annual infection rate associated with primary TKA has been reported to be approximately 0.6–2% [6, 7].

Infection following TKA is often accompanied by synovitis, and a specific pattern of synovitis has been reported to be a characteristic sign of infected TKA [8–10]. Although specific treatment strategies may differ depending on the type and causative microorganism of infection [11–13], synovectomy is an essential surgical procedure in most revision surgeries. However, there is a paucity of published studies describing detailed synovectomy techniques.

To our knowledge, most surgeons perform synovectomy using conventional surgical instruments such as a rongeur, electric cautery, and surgical blades during revision surgeries for infected TKA. However, there are no dedicated instruments to effectively remove infected synovium. Moreover, conventional techniques have major limitations, including incomplete removal of contaminated tissue, bleeding due to excessive removal of tissue, and difficulty in approaching the narrow space in the joint. Therefore, we describe a novel open synovectomy technique using an arthroscopy shaver blade to effectively treat intra-articular synovitis in second-stage revision surgery to treat infected primary TKA.

## Surgical technique

### Open synovectomy technique using a shaver blade

**Fig. 1:**
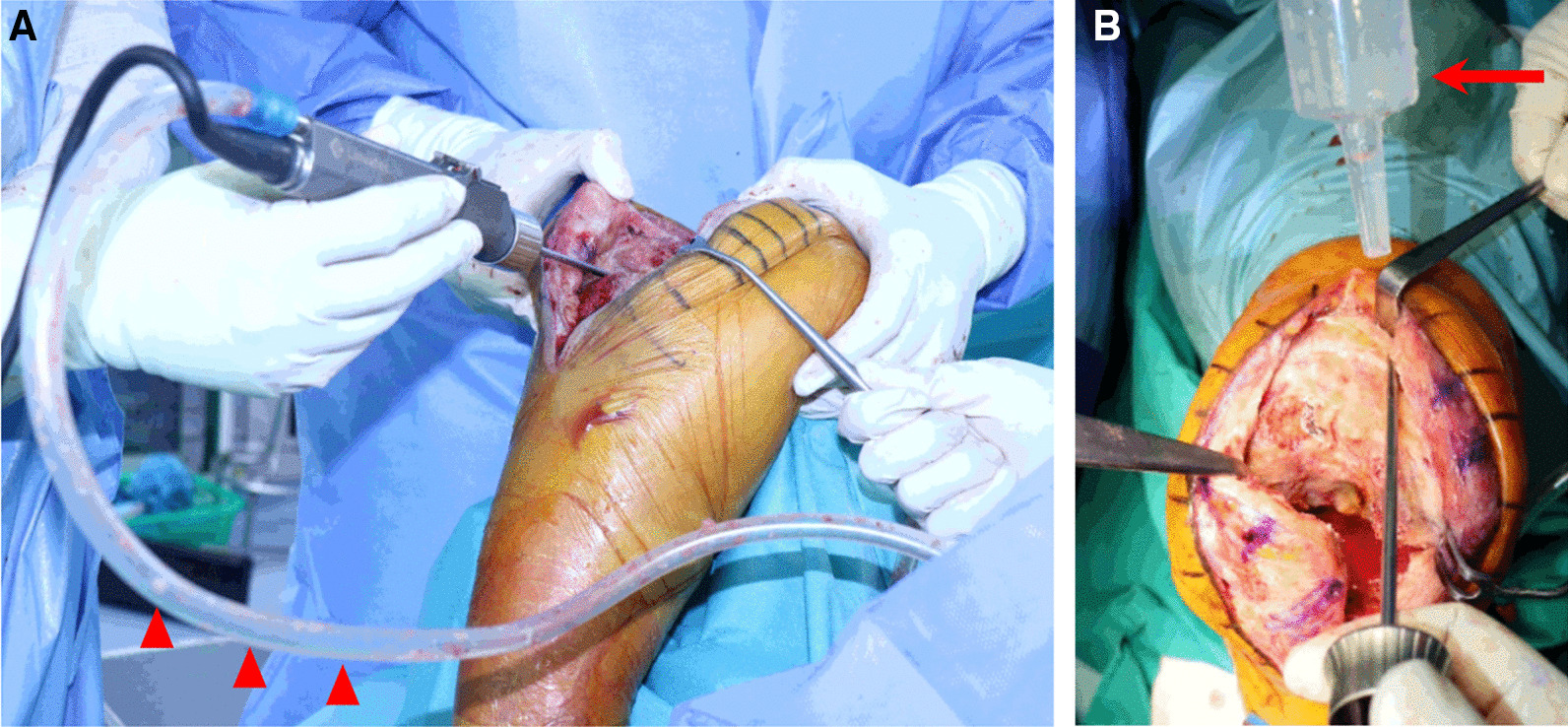
Basic supplementary preparations. **A** As the shaver blade rotates, the debrided tissue is aspirated through the central tube and collected in a suction trap (red-headed arrow). **B** During the shaving process, the assistant manually provides saline solution using an irrigation spoid (red arrow)

The patient is positioned supine on the operating table. The joint is exposed through conventional medial parapatellar arthrotomy. After removing pre-existing TKA components, synovectomy is performed. A 4.2-mm arthroscopy shaver blade (UltraFRR, CONMED, Largo, FL USA), powered by an arthroscopic power console, is assembled, and the handpiece is connected to suction drainage. Suction is supplied through the central cylinder of the shaver blade to bring the debrided fragments of soft tissue into the window. As the shaver blade rotates, the debrided tissue is aspirated through the central tube and collected in a suction trap (Fig. 1A). Unlike arthroscopic surgery, there is no need for a connection to an irrigation pump. After setting the speed to approximately 1800–3000 rpm, the surgeon can toggle between oscillating and forward modes. The shaver can be operated using its own buttons or pedals, as in arthroscopic surgery. During the shaving process, the assistant manually provides saline solution using an irrigation spoid (Fig. 1B).

**Fig. 2:**
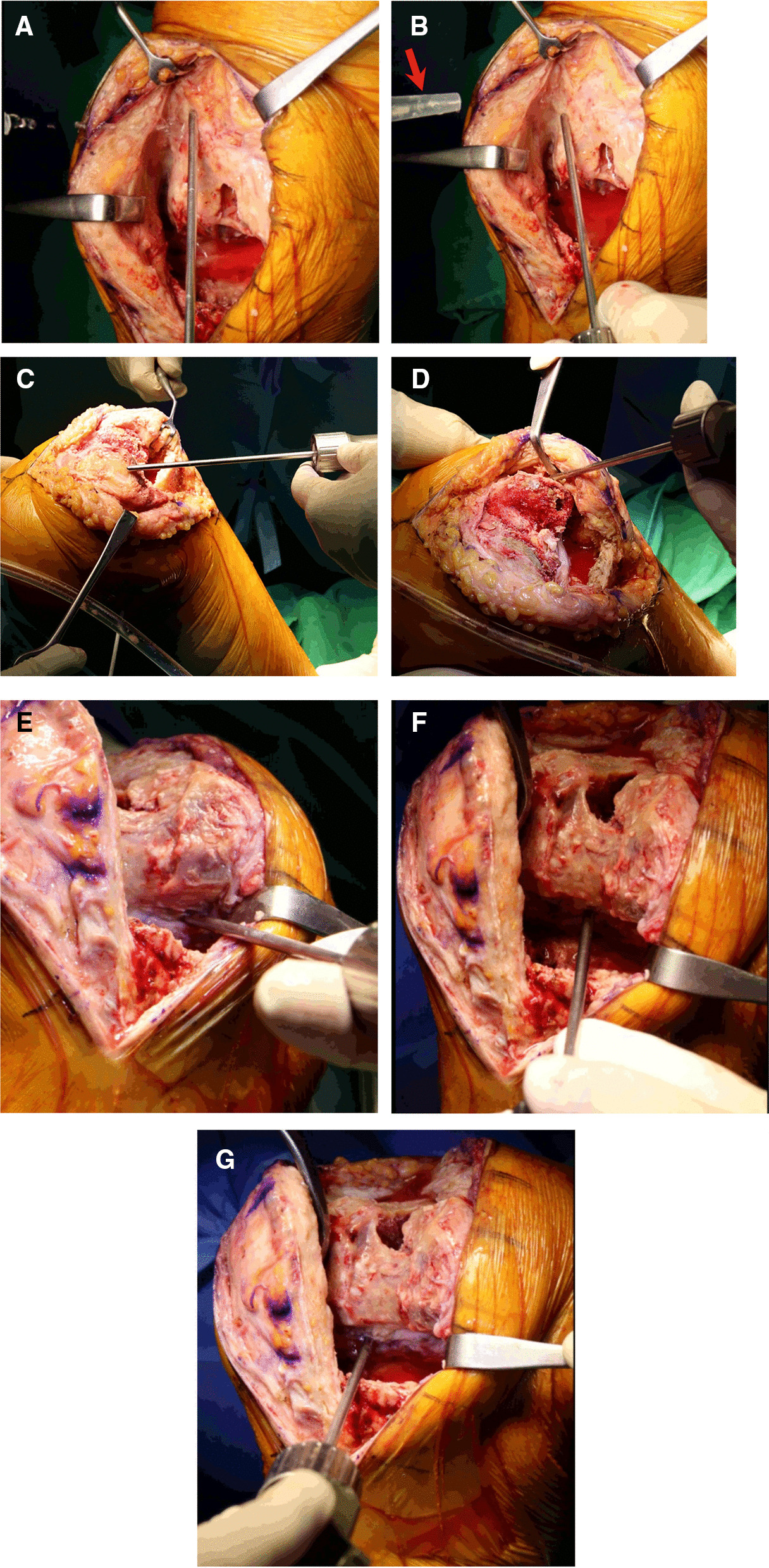
Detailed shaving process. **A**, **B** Anterior synovectomy is performed starting in the suprapatellar pouch. **C**, **D** The medial and lateral gutters are then visualized, and the synovitis is debrided. **E**, **F**, **G** With the knee joint in full flexion by the assistant, a shaver with a longer bar can be used to easily access the medial and lateral posterior compartments, which are generally difficult to access

**Fig. 3:**
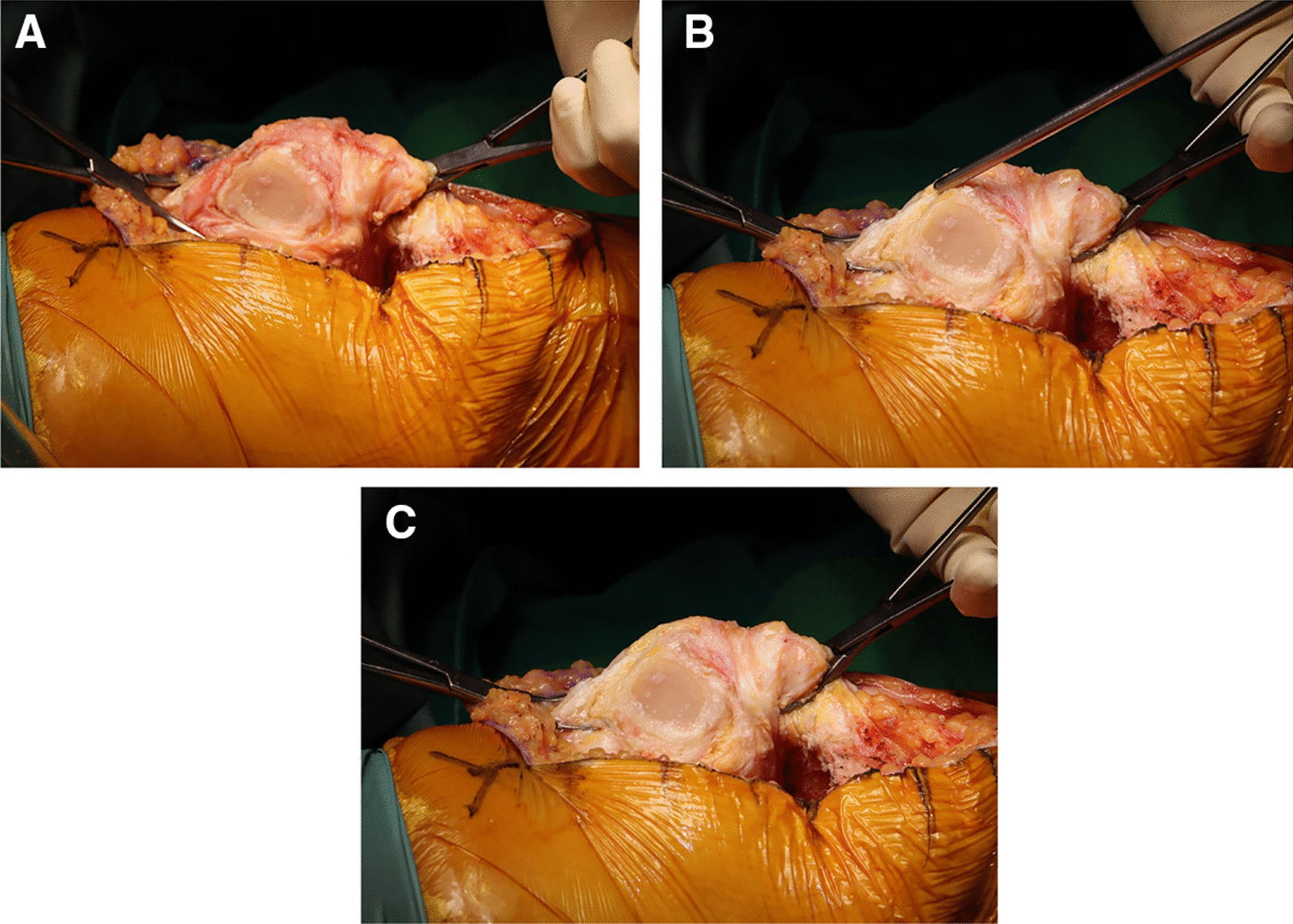
Synovectomy around the peri-patellar tissue. **A** Inflamed peri-patellar tissues are observed. **B** An open synovectomy using an arthroscopy shaver blade is performed. **C** After synovectomy, yellowish and healthy tissues are exposed

With direct visualization, the arthroscopy shaver blade is placed on the diseased synovial lining throughout the joint. Grossly inflamed, reddened, diseased synovium is debrided to reveal yellowish, healthy synovium. Anterior synovectomy is performed starting in the suprapatellar pouch (Fig. 2A, B). The medial and lateral gutters are then visualized, and the synovitis is debrided (Fig. 2C, D; Additional file 1: Video S1). While assistants maintain full flexion and traction of the knee joint, a shaver equipped with a longer bar can be used to easily access the medial and lateral posterior compartments, which are generally difficult to access (Fig. 2E–G). Finally, the inflamed soft tissues of the peripatellar area are debrided using the shaver (Fig. 3; Additional file 1: Video S1).

### Revision surgery and postoperative management

For all cases in second-stage revision TKA, the first surgery is performed using an antibiotic-loaded articulating cement spacer. A single closed suction drain is inserted and is removed 3‒7 days postoperatively. A knee immobilizer is applied to the knee at maximum extension. After drain removal, range of motion of the knee joint is permitted if pain subsides. One week postoperatively, partial weight-bearing with crutches is permitted. All patients undergo a 6-week course of organism-sensitive antibiotic therapy in consultation with an infectious disease specialist. Laboratory investigations, including serum white blood cell (WBC) count, erythrocyte sedimentation rate (ESR), and C-reactive protein (CRP), are periodically monitored to confirm a progressive decline in levels [14]. Second-stage re-implantation is performed only when there is sufficient clinical, radiographic, and laboratory evidence supporting eradication of the infection.

### Statistical analysis

The overall analysis was performed with SPSS software (version 25.0, SPSS Inc., Chicago, IL, USA). Comparisons of the mean value of serologic markers between two groups were investigated at each time point using Student’s *t* test with post *hoc* analysis by Bonferroni's method. The level of statistical significance was set at *p* < 0.05.

## Results

A total of 37 knees underwent second-stage revision surgery for the treatment of infected TKA between October 2018 and October 2020. Enrolled patients were followed up from the first-stage surgery [insertion of PROSTALAC (prosthesis of antibiotic-loaded acrylic cement)] [15] to at least 12 months after second-stage re-implantation surgery. This retrospective cohort study was approved by the Institutional Review Board of the authors’ hospital.

All surgeries were performed by a single surgeon at a single center. Outcomes of the conventional synovectomy technique using rongeur, electric cautery, and surgical blades (group A [*n* = 19 knees]) were compared with those of the novel synovectomy technique using arthroscopic shaver blade (group B [*n* = 18 knees]). Outcome measures included recurrence of infection; serological markers associated with infection, including WBC count, ESR, and CRP, during the follow-up period (first period, between the first- and second-stage surgery; second period, 6 weeks, and 3, 6, and 12 months after second-stage surgery); and the amount of bleeding through the suction drain was investigated.

**Fig. 4:**
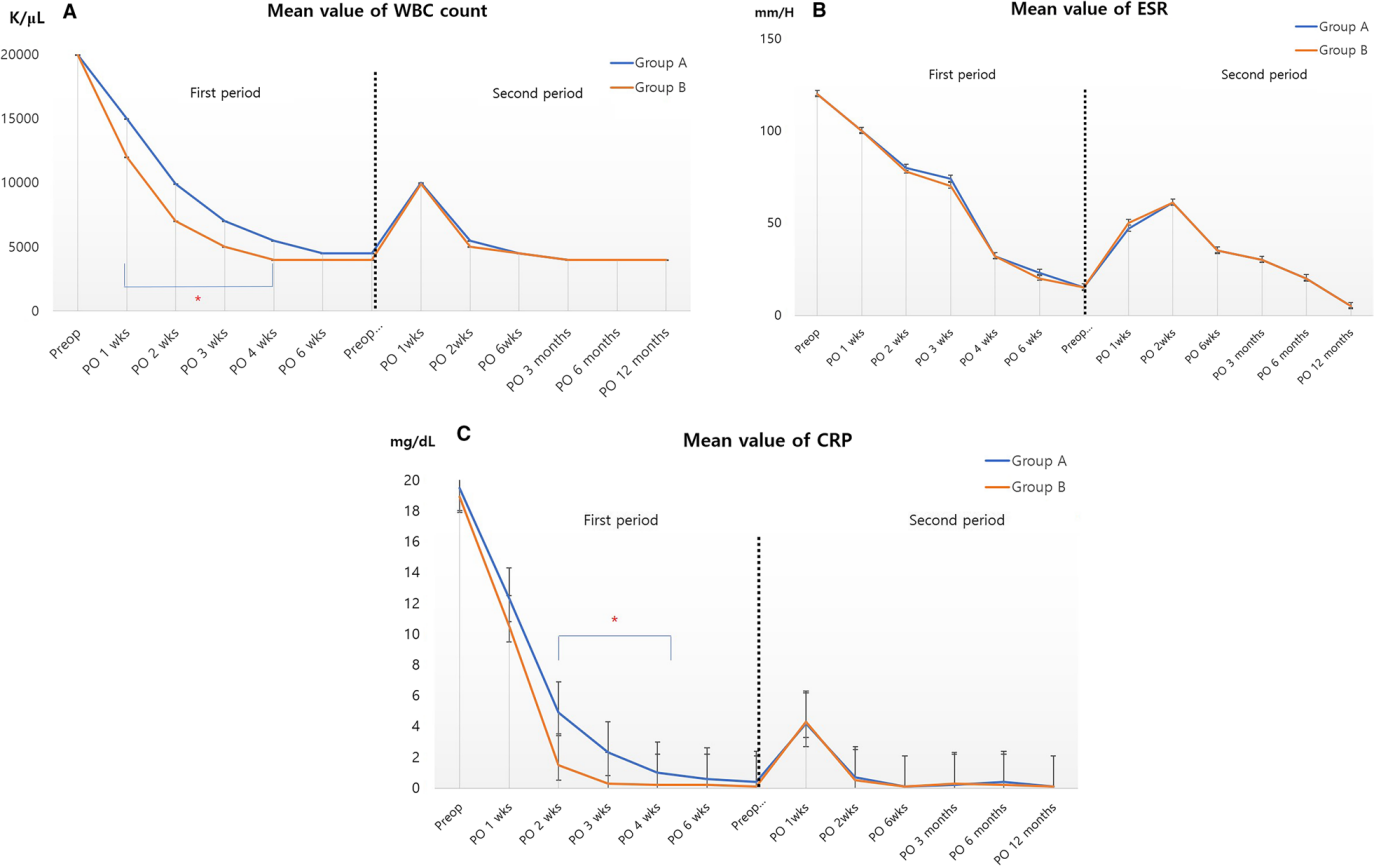
Serial changes of serologic markers, including **A** serum white blood cell (WBC) count, **B** erythrocyte sedimentation rate (ESR), and **C** C-reactive protein (CRP). These markers are periodically monitored to confirm a progressive decline in levels. The black dotted line indicates second-stage re-implantation.*Group B (i.e., novel technique) improved significantly faster in terms of WBC count and CRP level during the first period. The significant differences of the serologic markers were investigated with pairwise between -group and within-group comparisons, which were conducted using Student’s *t* test with post *hoc* analysis by Bonferroni's method. The level of statistical significance was set at *p* < 0.05

Demographic characteristics of the patients and outcomes during follow-up are summarized in Table 1. There was no recurrence of infection in either group; however, group B (i.e., novel technique) improved significantly faster in terms of WBC count and CRP level during the first period (Fig. 4). Meanwhile, the amount of bleeding through the suction drain was greater in group B, although the difference was not statistically significant (Table 2).

**Table 1:** Patients’ demographic characteristics

Variables	Total (*n* = 37)	Group A (*n* = 19)	Group B (*n* = 17)	*p* value
Mean age (years)^a^	72.9 (57.0–83.0)	72.1 (62.0–81.0)	73.8 (57.0–83.0)	0.847
Sex^b^				
Female	29 (78.4%)	29 (78.4%)	29 (78.4%)	
Male	8 (21.6%)	8 (21.6%)	8 (21.6%)	
BMI (kg/m^2^)^a^	25.0 (19.8–33.8)	25.0 (19.8–33.8)	25.0 (19.8–33.8)	0.865
Mean F/U period (months) ^a^	17.5 (12.0–37.0)	17.1 (12.0–34.0)	17.9 (12.0–37.0)	0.641
Diabetes mellitus ^b^	6/37 (16.2%)	3/19 (15.8%)	3/17 (17.6%)	0.881

*BMI* body mass index, *F/U* follow-up

^a^Values are given as means (ranges)

^b^Values are given as numbers (percentage)

**Table 2:** Outcomes

Variables	Total (*n* = 37)	Group A (*n* = 19)	Group B (*n* = 17)	*p* value
Drainage after first-stage surgery (ml)^a^	452.1 (80.0–650.0)	450.5 (120.0–650.0)	461.3 (80.0–620.0)	0.172
Mean duration of IV antibiotics (days) ^a^	32.5 (24.0–36.0)	33.1 (28.0–35.0)	33.1 (24.0–35.0)	0.381
Mean duration of oral antibiotics (days)^a^	14.7 (10.0–21.0)	14.1 (10.0–21.0)	15.1 (14.0 – 21.0)	0.632
Mean duration between 1st- and 2nd-stage surgery (days)^a^	43.5 (31.0–91.0)	44.6 (42.0–62.0)	42.8 (31.0–60.0)	0.109
Recurrence of infection	–	–	–	–

The amount of bleed through suction drain was the total amount before removal. At the surgeon's discretion, the drain was removed 3‒7 days postoperatively

*IV* intravenous

^a^Values are given as means (ranges)

## Discussion

The novel technique described in the present article was prompted by the need to facilitate arthroscopic access to the posterior compartment, which is difficult to access and clean using existing open surgical instruments such as rongeur, electric cautery, and/or surgical blades. We used a shaver blade commonly used in conventional arthroscopic surgery for open revision surgery to treat infected TKA.

Among patients who underwent surgery using this technique, serological markers associated with acute infection, including WBC count and CRP level, improved significantly faster during the follow-up period because more grossly contaminated or diseased tissue was effectively debrided using a shaver blade. In particular, meticulous debridement of infected tissues around the posterior compartment, which is difficult to access using conventional surgical instruments, was possible using a shaver equipped with a long bar (Fig. 3). Such rapid improvement in laboratory parameters may affect the prognosis of revision surgery for the treatment of infected TKA, such as reducing the possibility of recurrent infection, shortening the period of antibiotic use, and facilitating earlier recovery [14, 16, 17].

However, ESR decreased with a similar trend in both groups during the follow-up period. Because ESR has a longer half-life than CRP [18], it is more indicative of a persistent, deep-seated joint disease process [16, 19]. Using this novel technique, faster recovery of acute serological markers was achieved in revision surgery for infected TKA, which could help in the rapid improvement of infection.

Another advantage was that precise debridement was possible based on the surgeon’s intention. Conventional instruments, such as rongeur, electric cautery, and surgical blade, are unable to meticulously remove infected synovium; moreover, excessive tissue removal could cause bleeding or damage to healthy tissue. On the other hand, when using an arthroscopy shaver blade, it was possible to precisely debride the inflamed layer to reveal the healthy yellowish layer. It enables controlled management of the soft tissue [20]. In addition, this technique can be applied to various surgical scenarios for infected TKA, such as polyethylene exchange, first- and second-stage revision(s), as well as the situation described in this article.

For successful application of this technique, the shaver handpiece must be connected to the suction drain, and saline solution must be continuously supplied by an assistant (Fig. 1). The assistant should manually provide the saline solution using an irrigation spoid during surgery. As with arthroscopic surgery, a slower oscillating mode is usually useful for tissue removal [20]. This enables efficient removal of the debrided (i.e., infected) tissue.

Although this technique is relatively simple and efficient, excessive removal of infected tissue may result in large amounts of bleeding. In the present study, although the difference was not statistically significant, it was found that the amount of bleeding through the suction drain was larger in the group in which the shaver blade was used. This may be considered to be a limitation of using a shaver blade in the reduced field of direct view with the naked eye rather than the magnified screen used in conventional arthroscopy. Therefore, when performing open debridement of infected tissue rather than through arthroscopy, careful attention should be devoted to minimize the risk for unintended tissue or shaver-related damage [20].

Nevertheless, this technique is a novel example of applying arthroscopic instruments to open surgery and was an effective method to overcome the limitations associated with the use of conventional surgical instruments.

## Conclusions

A novel synovectomy technique using an arthroscopy shaver blade yielded favorable outcomes compared with the conventional technique. In particular, it may be an alternative method to improve the approach to the posterior joint space, which is often difficult to access.

## Supplementary Information

**Additional file 1:** Open synovectomy procedures using an arthroscopic shaver blade.

## Data Availability

Not applicable.

## Abbreviations

TKA: Total knee arthroplasty
WBC: Serum white blood cell
ESR: Erythrocyte sedimentation rate
CRP: C-reactive protein
PROSTALAC: Prosthesis of antibiotic-loaded acrylic cement
